# Synthesis and Evaluation of Antimicrobial Activity of [R_4_W_4_K]-Levofloxacin and [R_4_W_4_K]-Levofloxacin-Q Conjugates

**DOI:** 10.3390/molecules22060957

**Published:** 2017-06-08

**Authors:** Neda Riahifard, Kathy Tavakoli, Jason Yamaki, Keykavous Parang, Rakesh Tiwari

**Affiliations:** 1 Center for Targeted Drug Delivery, Department of Biomedical and Pharmaceutical Sciences, Chapman University School of Pharmacy, Harry and Diane Rinker Health Science Campus, Irvine, CA 92618, USA; riahi103@mail.chapman.edu (N.R.); tavak103@mail.chapman.edu (K.T.); 2 Department of Pharmacy Practice, Chapman University School of Pharmacy, Harry and Diane Rinker Health Science Campus, Irvine, CA 92618, USA; yamaki@chapman.edu

**Keywords:** amphiphilic cyclic peptide, conjugation, combination, levofloxacin, levofloxacin-Q, methicillin-resistant *Staphylococcus aureus*, *Klebsiella pneumonia*

## Abstract

The development of a new class of antibiotics to fight bacterial resistance is a time-consuming effort associated with high-cost and commercial risks. Thus, modification, conjugation or combination of existing antibiotics to enhance their efficacy is a suitable strategy. We have previously reported that the amphiphilic cyclic peptide [R_4_W_4_] had antibacterial activity with a minimum inhibitory concentration (MIC) of 2.97 µg/mL against Methicillin-resistant *Staphylococcus aureus* (MRSA). Herein, we hypothesized that conjugation or combination of the amphiphilic cyclic peptide [R_4_W_4_] with levofloxacin or levofloxacin-Q could improve the antibacterial activity of levofloxacin and levofloxacin-Q. Fmoc/tBu solid-phase chemistry was employed to synthesize conjugates of [R_4_W_4_K]-levofloxacin-Q and [R_4_W_4_K]-levofloxacin. The carboxylic acid group of levofloxacin or levofloxacin-Q was conjugated with the amino group of β-alanine attached to lysine in the presence of 2-(1*H*-benzotriazol-1-yl)-1,1,3,3-tetramethyluronium hexafluorophosphate (HBTU) and *N*,*N*-diisopropylethylamine (DIPEA) for 3 h to afford the products. Antibacterial assays were conducted to determine the potency of conjugates [R_4_W_4_K]-levofloxacin-Q and [R_4_W_4_K]-levofloxacin against MRSA and *Klebsiella pneumoniae*. Although levofloxacin-Q was inactive even at a concentration of 128 µg/mL, [R_4_W_4_K]-levofloxacin-Q conjugate and the corresponding physical mixture showed MIC values of 8 µg/mL and 32 µg/mL against MRSA and *Klebsiella pneumonia*, respectively, possibly due to the activity of the peptide. On the other hand, [R_4_W_4_K]-levofloxacin conjugate (MIC = 32 µg/mL and MIC = 128 µg/mL) and the physical mixture (MIC = 8 µg/mL and 32 µg/mL) was less active than levofloxacin (MIC = 2 µg/mL and 4 = µg/mL) against MRSA and *Klebsiella pneumoniae*, respectively. The data showed that the conjugation of levofloxacin with [R_4_W_4_K] significantly reduced the antibacterial activity compared to the parent analogs, while [R_4_W_4_K]-levofloxacin-Q conjugate was more significantly potent than levofloxacin-Q alone.

## 1. Introduction

The discovery of penicillin [1,2] and streptomycin [3] and other antibiotics led to the treatment of a number of prevalent microbial diseases. Resistance development emerged due to improper use and limited the application of these gold standard antibiotics and generated an urgent need for exploration of new antibiotics [4,5]. The decline in the numbers of marketed antibiotics was mainly due to the difficulty of compounds to penetrate bacterial cells [6] and high costs related to drug-development [7]. Modification, conjugation or combination of existing antibiotics to enhance the efficacy are among the suitable strategies. Modification of known antibiotics has been used to improve absorption, distribution, metabolism, and elimination (ADME) whereas conjugation of antibiotics with antibodies provides targeted delivery and release of antibiotics to the site of action [8,9]. Similarly, combination approaches have been applied to demonstrate improved antimicrobial therapy [10]. Furthermore, using two antibiotics with synergistic activity simultaneously can reduce the required dosage and toxicity of each compound. It has been previously shown that the combinational therapy broadens the antibacterial spectrum and lowers side effects. For example, a cationic antimicrobial peptide, arenicin-1, was able to enhance fluoroquinolone entry into the bacterial cytoplasmic membrane [11]. Also, a combination of antibiotics and antimicrobial cationic peptide brevinin-2CE improves bactericidal activity against multidrug-resistant bacteria [12].

Levofloxacin (**1**) is a well-known clinically used broad-spectrum fluoroquinolone antibiotic that is effective against Gram-positive, Gram-negative, and atypical bacteria [13]. This fluoroquinolone derivative contains a 6-fluoro substituent and 7-piperazinyl substituent on the quinolone ring structure with activity against the Gram-positive and atypical organisms [13,14]. Until now, levofloxacin has been an appropriate choice for community-acquired pneumonia (CAP), acute bacterial sinusitis (ABS), urinary tract infections (UTI), and pyelonephritis [15]. Levofloxacin inhibits bacterial topoisomerase IV and DNA gyrase [16]. Fluoroquinolones are hydrophilic in nature which reduces their ability to pass through bacterial lipopolysaccharides. Furthermore, the concentration of the fluoroquinolones in the bacterial cells is highly dependent on influx through porins [17].

**Figure 1:**
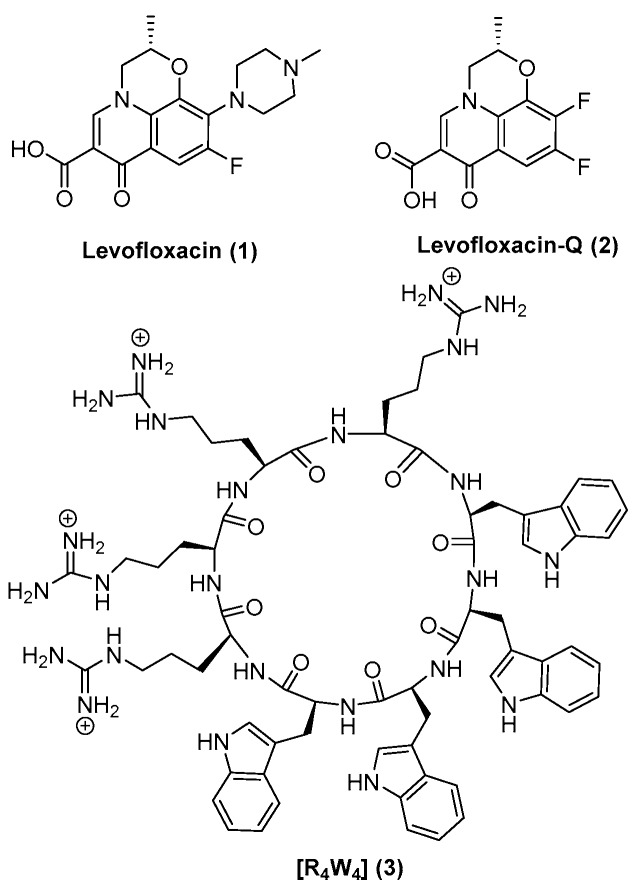
Chemical structures of levofloxacin (**1**), levofloxacin-Q (**2**), and amphiphilic cyclic peptide [R_4_W_4_] (**3**).

Antimicrobial peptides (AMPs) are vital parts of the innate immune system, which usually act at the level of the plasma membrane of the bacterial cell [18,19]. AMPs have a unique mode of action, which is mostly related to their cationic and amphipathic properties, making them capable of interacting with microbial membranes [20,21,22]. Accordingly, AMPs have attracted increasing attention as potential candidates against resistant bacteria [23,24]. Our group previously reported that the amphiphilic cyclic peptide [R_4_W_4_] (**3**) containing arginine (R) and tryptophan (W) residues (Figure 1) had antibacterial activity against MRSA with a MIC value of 2.97 µg/mL and showed bactericidal property when used in combination with known antibiotic tetracyclic [25]. Therefore, we hypothesized that conjugation or combination of the amphiphilic cyclic peptide [R_4_W_4_] (**3**) with levofloxacin (**1**) and levofloxacin-Q (**2**) could improve the antibacterial activity of **1** and **2** by enhancing their ability to penetrate through bacterial lipopolysaccharides and or by providing a synergistic effect with multiple mechanisms of action (Figure 1). Herein, we report the synthesis and evaluation of the antimicrobial activity of conjugates [R_4_W_4_K]-levofloxacin-Q (**9**) and [R_4_W_4_K]-levofloxacin (**8**) and compare it with their parent analogs and their physical mixtures, respectively.

## 2. Results

### 2.1. Chemistry

**Scheme 1:**
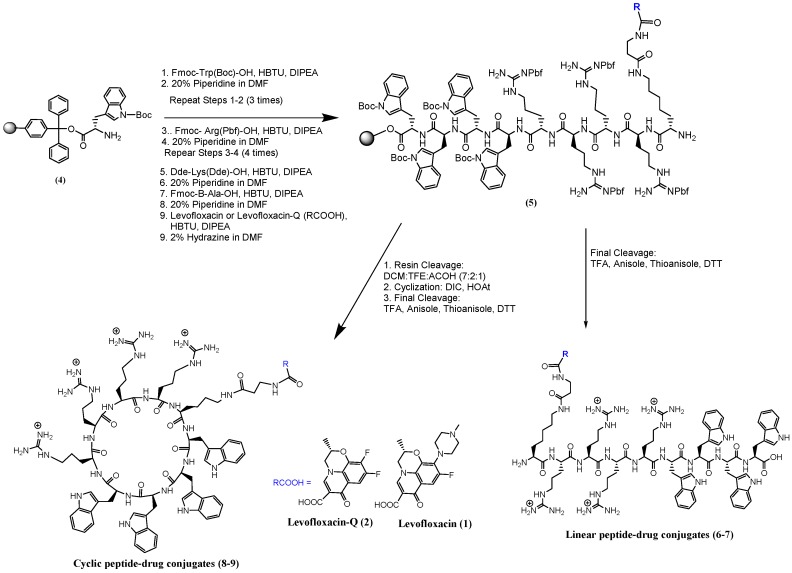
Synthesis of linear (R_4_W_4_K)-levofloxacin-Q (**7**), (R_4_W_4_K)-levofloxacin (**6**), cyclic [R_4_W_4_K]-levofloxacin-Q (**9**), and [R_4_W_4_K]-levofloxacin (**8**) conjugates.

**Figure 2:**
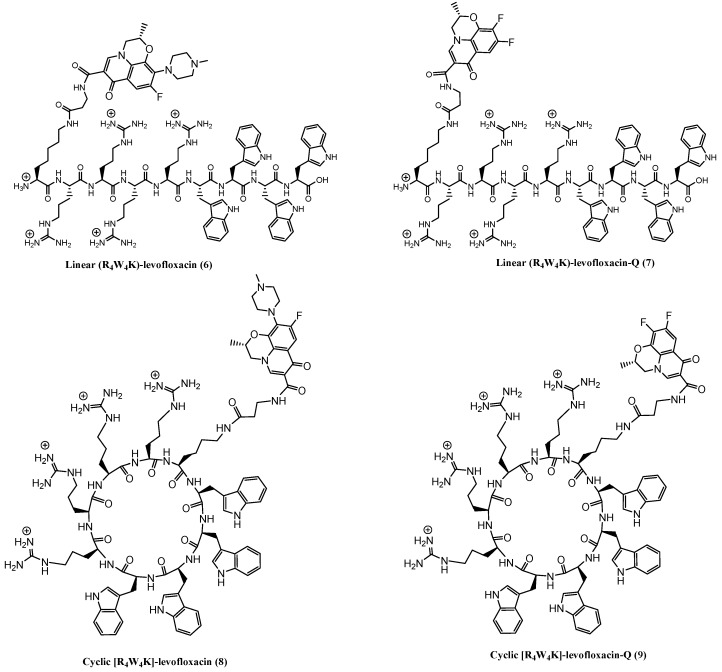
Chemical structures of cyclic and linear conjugate of [R_4_W_4_K] with levofloxacin and levofloxacin-Q.

Two cyclic peptide conjugates, two corresponding linear peptide conjugates (Scheme 1 and Figure 2), and cyclic peptide [R_4_W_4_] (Figure 1) were synthesized by using 9-fluorenylmethyloxy-carbonyl (Fmoc)/tBu solid-phase peptide synthesis. The synthesis of amphiphilic cyclic peptide [R_4_W_4_] (**3**) has been previously reported [25] and the same protocol was used with minor modifications. H-Arg(Pbf)-2-chlorotrityl resin was used to assemble arginine residues first followed by tryptophan to yield (**3**).

To synthesize conjugates of **1** or **2**, a lysine residue was incorporated into the cyclic peptide followed by a short β-alanine linker. For the synthesis of conjugated peptides, Trp(Boc)-2-chlorotrityl resin was used as the solid-phase resin. Fmoc-Arg(Pbf)-OH, Fmoc-Trp(Boc)-OH, Fmoc-β-alanine-OH, and Fmoc-Lys(Dde)-OH were used as building block amino acids in the synthesis. The peptide sequence KRRRRWWWW was first assembled on solid-phase using Fmoc/tBu solid-phase methodology. Fmoc-β-alanine in the side chain of lysine was attached to Dde protected lysine at N-terminal. After the deprotection of the Fmoc group of β-alanine, the free NH_2_ group was used for conjugation with levofloxacin or levofloxacin-Q using coupling and activating agents, HBTU and DIPEA, respectively. The conjugation was completed in 3 h by monitoring with MALDI mass spectroscopy. The N-terminal protection of the Dde group in the lysine was deprotected using 2% hydrazine hydrate in *N,N*-dimethylformamide (DMF). The resin was divided into two sections.

The first section was used for the synthesis of linear peptide–drug conjugate. The linear peptide–drug conjugate was cleaved using freshly prepared cleavage cocktail of trifluoroacetic acid (TFA), anisole, thioanisole, and dithiothreitol (DTT) followed by precipitation and purification.

The second part of the resin was used for the synthesis of cyclic peptide–drug conjugate. The cleavage of the protected peptide from the resin was accomplished by using cleavage cocktail, dichloromethane, trifluoroethanol, acetic acid (DCM:TFE:AcOH; 7:2:1 *v*/*v*/*v*) to generate the side-chain protected linear peptide. The N- to C-terminal cyclization was carried out using *N*,*N*′-diisopropylcarbodiimide (DIC), 1-hydroxy-7-azabenzotriazole (HOAT) in the presence of DCM/DMF overnight. Once the peptide became cyclized as confirmed by MALDI, the crude peptide was cleaved using freshly prepared final cleavage cocktail of TFA, anisole, thioanisole, and DTT followed by precipitation and purification. After final deprotection of the side chains, HPLC and MALDI were used for purification and characterization of the compounds, respectively. The physical mixtures of the peptides and drug were prepared in 1:1 molar ratio. 

### 2.2. Antibacterial Assay

MIC values for all four conjugated peptides were measured for activity against *Klebsiella pneumoniae* and MRSA bacterial strains (Table 1) and compared with physical mixtures of levofloxacin (**1**) + [R_4_W_4_] (**3**) and levofloxacin-Q (**2**) + [R_4_W_4_] (**3**). Although levofloxacin-Q (**2**) was inactive even at a concentration of 128 µg/mL, [R_4_W_4_]-levofloxacin-Q (**9**) and the corresponding physical mixture showed MIC values of 8 µg/mL, possibly due to the activity of the peptide. On the other hand, [R_4_W_4_K]-levofloxacin (**8**) (MIC = 32 µg/mL) and the physical mixture levofloxacin + [R_4_W_4_] (MIC = 8 µg/mL) were less active than levofloxacin (**1**) (MIC = 2 µg/mL).

The evaluation of antibacterial activity of [R_4_W_4_K]-levofloxacin (**8**) demonstrated that the conjugation of levofloxacin (**1**) reduced the activity of the lead compound and did not show synergistic activity. However, [R_4_W_4_K]-levofloxacin-Q (**9**) and levofloxacin-Q (**2**) + [R_4_W_4_] (**3**) showed higher antibacterial activity compared with levofloxacin-Q (**2**) that can be explained by the possible antibacterial activity of **3**. These data indicate that the presence of both the antibacterial peptide and levofloxacin in [R_4_W_4_K]-levofloxacin (**8**) do not generate any synergistic antibacterial effect, possibly due to the incomplete hydrolysis of [R_4_W_4_K]-levofloxacin to levofloxacin and [R_4_W_4_K(β-alanine)] and masking the pharmacophore carboxylic acid group of levofloxacin in the conjugate. Reduced antibacterial activity of the physical mixture by two-fold can be attributed to the dilution of [R_4_W_4_] after mixing with the levofloxacin in 1:1 molar ratio or physical interaction of the cyclic peptide with levofloxacin.

## 3. Discussion

Our laboratory has previously reported that the amphiphilic cyclic peptide [R_4_W_4_] (**3**), a cell-penetrating peptide (CPP) (Figure 1) containing arginine (R) and tryptophan (W) residues has antibacterial activity with MIC value of 2.97 µg/mL against MRSA, a multidrug-resistant bacterium commonly found in the hospital and community settings [25]. Twenty-four-hour time-kill studies that evaluated **3** in combination with tetracycline demonstrated bactericidal activity at 4–8 times the MIC of tetracycline against MRSA (MIC = 0.5 µg/mL) and 2–8 times the MIC against *Escherichia coli* (MIC = 2 µg/mL). This study suggested that amphiphilic cyclic CPPs with antibacterial activity can be used in combination with tetracycline to provide a significant benefit against multidrug-resistant pathogens when compared with the antibiotic treatment alone. The mechanism of cellular uptake indicated that the intracellular transportation of amphiphilic cyclic peptide [R_4_W_4_] (**3**) was controlled by several mixed pathways [26]. Herein, we intend to see the effect of covalent conjugation or a physical mixture of the peptide with another antibiotic, levofloxacin (**1**), against MRSA and *Klebsiella pneumoniae*.

Levofloxacin (**1**) is a well-known broad-spectrum antibiotic, which is active against both Gram-positive and Gram-negative bacteria and is commonly is used for the treatment of several types of infection in the clinical setting. It inhibits an important enzyme, topoisomerase, which is required by the bacterial cell for DNA cell division. Levofloxacin-Q acid (**2**) is a derivative of levofloxacin which is inactive due to absence of piperazinyl moiety at C-7. These two compounds were used in this study for covalent and physical mixture with the amphiliphic cyclic peptide [R_4_W_4_] (**3**). 

The attachment of (**1**) or (**2**) was achieved through a linker lysine-β-alanine to provide sufficient space between the drug and cyclic peptide **3**. The synthesis was designed to minimize the structural changes. A lysine residue was incorporated into the sequence of the peptide containing four tryptophan and four arginine residues. The cyclic amphiphilic peptide was synthesized using a minor modification to our reported procedure [25] with the change of the resin to arginine(Pbf)-2-chlorotrityl resin. The peptide was purified by HPLC and analyzed by using MALDI mass spectroscopy and used in the antibacterial assay. 

The synthesis of conjugates of **1** and **2** was accomplished through Fmoc/tBu solid-phase synthesis (Scheme 1). The carboxylic acid functional group of **1** and **2** (Figure 1) was used for conjugation with the amino group in the cyclic peptide. The conjugation strategy was carried out on the solid-support for quick and fast access to the conjugated compound as compared to the solution-phase chemistry that requires multiple protection/deprotection steps with tedious purification at each step. To facilitate solid-phase synthesis, cyclic peptide synthesis was started using Trp(Boc)-2-chlorotrityl resin followed by **3** coupling of Fmoc-Trp(Boc)-OH, **4** coupling of Fmoc-Arg(Pbf)-OH, one coupling of Dde-Lys(Fmoc)-OH, and one coupling of Fmoc-β-alanine-OH to assemble the protected peptide W(Boc)_4_R(Pbf)_4_K-β-alanine-NH_2_ on the solid support. Coupling was carried out in the presence of HBTU and DIEPA in anhydrous DMF and deprotection was accomplished using 20% piperidine in DMF. The conjugation of **1 **or **2 **was achieved using HBTU/DIPEA for 3 h after monitoring with MALDI mass spectroscopy. The Dde group was removed to provide free N-terminal using 2% hydrazine hydrate. The linear conjugated peptide of **1 **or **2 **was achieved after final cleavage of the peptide from the side chain and resin using a cleavage cocktail followed by purification and analysis. The cyclization was facilitated for cyclic levofloxacin or levofloxacin-Q conjugated peptides using a cocktail, DCM:TFE:AcOH followed by cyclization overnight using DIC/HOAt in DCM:DMF. The cyclic peptides were cleaved and deprotected followed by HPLC purification to afford conjugated peptides.

The antibacterial activity of linear or cyclic conjugated peptides of levofloxacin or levofloxacin-Q were tested against MRSA and *Klebsiella pneumoniae* (Table 1). The bacterial strains were obtained from our local community. Meropenem and vancomycin were used as control antibiotics along with **1** and **2 **in the antibacterial assay. The physical mixture of levofloxacin (**1**) + [R_4_W_4_] (**3**) was more potent than the covalent conjugate of antibiotic and peptide, presumably because of the presence of the free parent analogs. Furthermore, the physical mixture (Levofloxacin-Q + [R_4_W_4_]) and or covalent conjugation of levofloxacin-Q ([R_4_W_4_]-Levofloxacin-Q) showed improved activity when compared with levofloxacin-Q. Levofloxacin-Q alone showed antibacterial activity at a concentration higher than 128 µg/mL against both MRSA and *Klebsiella pneumoniae* which was improved in the physical mixture/covalent conjugation to 8 and 32 µg/mL against MRSA and *Klebsiella*, respectively. However, the antibacterial activity of the physical mixture (**1 **+ **3**) or covalently conjugated compound ([R_4_W_4_]-levofloxacin (**8**)) was reduced when compared to **1 **or **3** alone. Similarly, the conjugate of the linear peptide (R_4_W_4_-levofloxacin (**6**)/R_4_W_4_-levofloxacin-Q (**7**)) was also tested which showed low antibacterial activity (MIC = 64 µg/mL) as compared to the cyclic peptide (**3**) and or levofloxacin (**1**) alone. We assume that conjugation of levofloxacin (**1**) using the COOH group blocks the pharmacophore group of the drug. The conjugated compound needs to be hydrolyzed to release the drug-containing free COOH group. The amide linkage of the drug with the peptide possibly does not hydrolyze fast in the assay conditions. The reduced antibacterial activity of the physical mixture (1:1 molar ratio) is possibly due to the presence of less cyclic peptide or possible physical interaction through electrostatic interactions of the cyclic peptide and levofloxacin or levofloxacin-Q. It was well documented that the C-7 position in fluoroquinolones antibacterial agents associated with their antibacterial spectrum, bioavailability, and safety [27,28,29]. The bulkier groups were found to be more favored as compared to the smaller group (fluorine) on C-7 substituent [30,31]. Therefore, a fluoroquinolone with a C-7 substituent containing a piperazinyl ring with the N4 methyl group was found to be more potent.

The discovery and development of novel antibiotics have been limited during the last few years. The discovery of novel antibiotics is urgently required to treat antibiotic-resistant bacteria. One of the emerging areas is using antibody–antibiotic conjugates (AAC), which have shown potential in recent years against killing the resistant bacteria, such as MRSA [8]. However, due to the bulky nature of the antibody and the associated immunogenic response by the body, this strategy will pose more challenges as compared to the use of peptide–antibiotic conjugates (PAC). Thus, the conjugate of the amphiphilic cyclic peptide [R_4_W_4_] (**3**) with antibiotics can be an alternative strategy. However, structural optimization is required to ensure that the conjugates dissociate in order to release the antibiotic drug and cyclic peptide in such a way that they work synergistically. Another worthwhile approach in this direction is to examine the combination of more conventional antibiotics with the amphiphilic cyclic antibacterial peptide in combination strategies to exploit multiple mechanisms to kill resistant bacteria. 

## 4. Materials and Methods

### 4.1. General Methods

Protected L-amino acids and preloaded H-Trp(Boc)-2-chlorotrityl resin were purchased from Aapptec LLC, Louisville, KY, USA. All the other chemicals and reagents, such as DIPEA, piperidine, trifluoroacetic acid (TFA), DMF, and HPLC grade acetonitrile were purchased from Sigma-Aldrich Co (Milwaukee, WI, USA). Levofloxacin (TCI America, Portland, OR, USA) and Levofloxacin Q-acid were purchased from TCI America, USA. The chemical structures of final products were confirmed by high-resolution MALDI-TOF (GT 0264) from Bruker Daltonics, Inc., San Jose, CA, USA Final compounds were purified by a reversed-phase HPLC from Shimadzu Scientific Instruments, Inc., Pleasanton, CA, USA (LC-20AP) using a gradient system of acetonitrile and water using a reversed-phase preparative column (XBridge BEH130 Prep C18). Methicillin Resistant *Staphylococcus aureus* (Los Angeles County (LAC) clone) was obtained from the Los Angeles Public Health Department, CA, USA. Mueller Hinton media were purchased from Hardy Diagnostics, Lacey, WA, USA.

### 4.2. Synthesis of Cyclic Peptide [R_4_W_4_] *(**3**)*

The peptide was manually synthesized using a Chemglass peptide synthesis vessel (#CG1860) with the help of bubbling anhydrous nitrogen gas as reported by us previously—with minor modification. H-Arg(Pbf)-2-chlorotrityl resin (0.44 meq/g, 0.3 mmole, 681 mg) was added to the glass vessel and DMF (50 mL, 30 min × 2) with the help of nitrogen gas to swell the resin. Fmoc-Arg(Pbf)-OH (1.2 mmol, 778 mg) was coupled using HBTU (1.2 mmol, 455 mg), DIPEA (2.4 mmol, 1.0 mL) and DMF (15 mL) for 2 h. The resin was washed with DMF (25 mL × 3) and followed by deprotection of the Fmoc group using 20% piperidine in DMF (20 mL, 10 min × 2) with the washing of the resin at the end. Two more couplings were performed using Fmoc-Arg(Pbf)-OH followed by four successive couplings with Fmoc-Trp(Boc)-OH (1.2 mmol, 632 mg), HBTU (1.2 mmol, 455 mg), DIPEA (2.4 mmol, 1.0 mL), and DMF (15 mL) for 2 h. At the end, the N-terminal Fmoc group was deprotected followed by cleavage of the protected peptide from the resin by agitation with a cleavage cocktail, dichloromethane, trifluoroethanol, acetic acid (TFE:AcOH:DCM; 1:2:7, *v*/*v*/*v*, 50 mL) for 3 h. The filtrate was evaporated, and hexane (2 × 20 mL) and DCM (2 ×15 mL) were added to the residue to remove the acetic acid from the mixture. The crude material was solidified as a white solid. The crude peptide was dried under vacuum overnight followed by the addition of anhydrous DMF (200 mL), anhydrous DCM (50 mL), HOAt (1.2 mmol, 4 equiv, 136 mg) with stirring and dropwise addition of DIC (1.32 mmol, 4.4 equiv, 207 µL) to the crude protected linear peptide for cyclization. The reaction mixture was stirred under nitrogen overnight followed by MALDI analysis which confirmed complete cyclization. The solvents were removed under reduced pressure followed by complete deprotection of the peptide by agitation with freshly prepared cleavage cocktail reagent R using trifluoroacetic acid/anisole/1,2-ethanedithiol/thioanisole cocktail (90:2:3:5 (*v*/*v*/*v*/*v*), 20 mL) for 2 h at room temperature followed by centrifugation and precipitation to analyze the crude peptide. The crude peptide was purified by a reversed-phase high-pressure liquid chromatography (RP-HPLC) system with a gradient from 0 to 100% acetonitrile (CH_3_CN) containing 0.1% (*v*/*v*) TFA and water containing 0.1% (*v*/*v*) TFA for 1 h with a flow rate of 8.0 mL/min at a wavelength of 220 nm. [R_4_W_4_] (**3**): MALDI-TOF (*m*/*z*): C_68_H_88_N_24_O_8_: calcd., 1368.7217; found, 1369.4419 [M + H]^+^.

### 4.3. Synthesis of Linear (R_4_W_4_K)-Levofloxacin *(**6**)* and (R_4_W_4_K)-Levofloxacin-Q *(**7**)* and Cyclic [R_4_W_4_K]-Levofloxacin *(**8**)* and [R_4_W_4_K]-Q-Levofloxacin *(**9**)* Conjugates

First, the protected peptide W(Boc)_4_R(Pbf)_4_K-β-alanine-NH_2_ on the solid support was synthesized using Fmoc-based solid-phase peptide synthesis (Fmoc-SPPS) as described earlier, used for conjugation of levofloxacin and levofloxacin-Q acid. The synthesis was carried out using NH_2_-Trp(Boc)-2-chlorotrityl resin (loading, 0.3 mmol/g, 1 gm, 0.3 mmol) after swelling the resin with agitation in DMF (100 mL, 30 min × 2) and bubbling anhydrous nitrogen gas. Coupling and a deprotection cycle followed in order to assemble the peptide sequence on the solid support. Coupling of amino acids was performed using 4 equiv. of amino acids (three times coupling of Fmoc-Trp(Boc)-OH, four times coupling of Fmoc-Arg(Pbf)-OH, one coupling using Dde-Lys(Fmoc)-OH, and one coupling using Fmoc-β-Ala-OH) in the presence of HBTU (4 equiv.) and DIPEA (8 equiv.) in DMF (15 mL) as coupling and activating reagents, respectively, for 2 h. After every coupling, the resin was washed with DMF (20 mL × 3) followed by deprotection of the Fmoc group using 20% piperidine in DMF (*v*/*v*, 20 mL × 2, 10 min). The resin was washed with DMF (20 mL × 3). After the synthesis of the protected peptide on the resin, W(Boc)_4_R(Pbf)_4_K-β-alanine-NH_2_, the levofloxacin or Q-levofloxacin (4 equiv.) were conjugated in the presence of HBTU (4 equiv.) and DIPEA (8 equiv.) with agitation under nitrogen for 3 h. Small cleavage of the resin confirmed the formation of the conjugated peptide. The Dde protecting group was removed using 2% hydrazine hydrate in DMF (*v*/*v*, 30 mL, 10 min × 4) from the N-terminal of lysine to afford the free NH_2_ group. Half of the resin was washed with DMF (15 mL, 2 × 5 min) and used for linear peptide–drug conjugate by complete cleavage of peptidyl resin using a cleavage cocktail TFA, anisole, thioanisole, DDT (90:2:3:5, *v*/*v*/*v*, 20 mL) for 3 h followed by precipitation and centrifugation. The second half of the resin was used for cyclization using cleavage of the protected peptide from the resin with agitation in a cocktail (dichloromethane, trifluoroethanol, acetic acid (TFE:AcOH:DCM; 1:2:7, *v*/*v*/*v*, 50 mL)) for 3 h. The filtrate was evaporated, and hexane (2 × 20 mL) and DCM (2 × 15 mL) were added to the residue to remove the acetic acid from the mixture. The crude material was solidified as a white solid. The crude peptide was dried under vacuum overnight. Anhydrous DMF (200 mL), anhydrous DCM (50 mL), DIC (4.4 equiv.) and HOAt (4 equiv.) were added to the crude unprotected linear peptide for cyclization. The reaction mixture was stirred under nitrogen overnight. After the formation of the cyclic peptide was confirmed by MALDI analysis, the solvents were removed under reduced pressure. The crude peptide was dried overnight. The final cleavage cocktail (TFA, anisole, thioanisole, DTT, 90:2:3:5, *v*/*v*/*v*, 20 mL) was added to the crude product. The mixture was stirred at room temperature for 5 h. The crude peptide was precipitated in cold diethyl ether and centrifuged, purified by using reversed-phase HPLC, and lyophilized. The molecular mass of synthesized compounds are as follows: Linear (R_4_W_4_K)-levofloxacin (**6**): MALDI-TOF (*m*/*z*): C_95_H_125_FN_30_O_14_: calcd., 1928.9976; found, 1929.7300 [M + H]^+^, 1930.7400 [M + 2H]^+^; Linear (R_4_W_4_K)-levofloxacin-Q (**7**): MALDI-TOF (*m*/*z*): C_90_H_114_F_2_N_30_O_14_: calcd., 1848.9037; found, 1871.6920 [M + Na]^+^, 1889.6823 [M + K]^+^; cyclic [R_4_W_4_K]-levofloxacin (**8**): MALDI-TOF (*m*/*z*): C_95_H_123_FN_30_O_13_: calcd., 1910.9870; found, 1912.7700 [M + 2H]^+^; cyclic [R_4_W_4_K]-levofloxacin-Q (**9**): MALDI-TOF (*m*/*z*): C_90_H_112_F_2_N_30_O_13_: calcd., 1830.8932; found, 1832.4802 [M + 2H]^+^.

### 4.4. Antibacterial Assay

The antibacterial activities of synthesized peptides were evaluated against two bacterial strains and compared to a control antibiotic and [R_4_W_4_]. Gram-negative bacteria: *Klebsiella pneumoniae *(Clinical strain (levofloxacin-susceptible)) and the Gram-positive bacteria: Methicillin-Resistant *Staphylococcus aureus *(Los Angeles County (LAC) clone) were employed for the determination of antimicrobial activities of peptides. MRSA and *Klebsiella pneumoniae* were inoculated into 5 mL of* Mueller Hinton agar (MH)* at 37 °C and shaken in an orbital shaker at 175 rpm overnight. The cultured suspension was diluted in 5 mL normal saline until it achieved 0.5 McFarland (1.5 × 10^8 ^bacterial cell density) turbidity. An amount of 40 μL of the McFarland solution was added to 5980 μL of MH media to make 1/150 dilution. Most peptides were dissolved in distilled water (except some of them that were dissolved in 50 mM NH_4_HCO_3 _to improve the solubility) to make 256 μg/mL solutions. Minimal inhibitory concentrations (MICs) were determined using the broth microdilution method. Briefly, 200 μL of all tested peptides and controls was added in the first column of the 96-well plate. Then, by adding 100 μL of media to the other wells and performing a 2-fold dilution in each well, serial dilution of the compound was prepared. After a series of 2-fold dilutions, 100 μL of bacterial solution was added to all wells, and then the microliter plates were incubated statically at 37 °C overnight. MICs were determined as the minimal concentration at which no visible bacterial growth was present. All experiments were conducted in triplicate. 

## 5. Conclusions

In this study, we attempted to improve the antibacterial activity of levofloxacin by conjugating to [R_4_W_4_] (**3**), a cationic antibacterial peptide. The parent peptides and the final conjugates were successfully synthesized by Fmoc-SPPS, purified by HPLC, and characterized by MALDI. The physical mixture of [R_4_W_4_] (**3**) and levofloxacin-Q (**2**) (an inactive drug) and the conjugation showed improved antibacterial activity in comparison to the parent analog alone. On the other hand, the conjugation diminished the activity of **1**, while the corresponding physical mixture just reduced the activity. Further studies are required to explore antimicrobial peptides with synergistic activity with other antibiotics. These data provided insights into designing antibacterial-peptide-antibiotics and combinations to improve antimicrobial activity.

## Figures and Tables

**Table 1:** Antibacterial assay against Gram-positive and Gram-negative bacteria.

Compounds	MIC (μg/mL) ^1 ^	
	Methicillin-Resistant *Staphylococcus aureus* (MRSA)	*Klebsiella pneumoniae*
Meropenem	N/A ^2^	1
Vancomycin	1	N/A
Levofloxacin (**1**)	2	4
Levofloxacin-Q (**2**)	>128	>128
[R_4_W_4_] (**3**)	4	16
Conjugate cyclic [R_4_W_4_]-levofloxacin (**8**)	32	>128
Conjugate linear (R_4_W_4_)-levofloxacin (**6**)	64	>128
Levofloxacin (**1**) + [R_4_W_4_] (**3**)	8	32
Levofloxacin-Q (**2**) + [R_4_W_4_] (**3**)	8	32
Conjugate cyclic [R_4_W_4_]-Levofloxacin-Q (**9**)	8	32
Conjugate linear (R_4_W_4_)-Levofloxacin-Q (**7**)	ND ^3^	ND

^1 ^MIC measurement reflects triplicate of three experiments; ^2 ^N/A = not active; ^3 ^ND = not determined.
